# Dynamics of telomeric repeat-containing RNA expression in early embryonic cleavage stages with regards to maternal age

**DOI:** 10.18632/aging.103922

**Published:** 2020-08-29

**Authors:** Paweł Kordowitzki, Isabel López de Silanes, Ana Guío-Carrión, Maria A. Blasco

**Affiliations:** 1 Institute of Animal Reproduction and Food Research of Polish Academy of Sciences, Olsztyn, Poland; 2 Institute for Veterinary Medicine, Nicolaus Copernicus University, Torun, Poland; 3 Telomeres and Telomerase Group, Spanish National Cancer Research Centre (CNIO), Madrid, Spain

**Keywords:** reproductive aging, oocytes, TERRA, early embryonic development, telomere length

## Abstract

Telomeres are transcribed into long non-coding RNAs known as Telomeric Repeat-Containing RNA (TERRA). They have been shown to be essential regulators of telomeres and to act as epigenomic modulators at extra-telomeric sites. However the role of TERRA during early embryonic development has never been investigated. Here, we show that TERRA is expressed in murine and bovine early development following a wave pattern. It starts at 4-cell stage, reaching a maximum at the 16-cell followed by a decline at the morula and blastocyst stages. Moreover, TERRA expression is not affected by increasing oocyte donor age whereas telomere length does. This indicates that TERRA expression is independent of the telomere length in early development. Our findings anticipate an essential role of TERRA in early stages of development and this might be useful in the future for a better understanding of age related female infertility.

## INTRODUCTION

Due to many social and economic factors, women in the last decade postpone their first pregnancy until an advanced age [1], and the increased maternal age has resulted in sub- and infertility [2], as well as in increased oocyte aneuploidy [3], and pregnancy complications [4–9]. Proposed mechanisms for age related decline in the female reproductive axis involve neuroendocrine deficiency [10], mitochondrial dysfunction [11], as well as epigenetic modifications. Telomere homeostasis has also been shown to play a critical role during these stages as indicated by alterations in oocyte chromosome telomere length and meiosis abnormalities [9, 12]. Oocyte dysfunction is linked to two main phenomena: reduced formation of chiasmata during fetal oogenesis, and accumulation of reactive oxygen damage during the prolonged interval from birth until ovulation. Late exit from the developmental line during oogenesis presumably contributes to the first phenomenon what would further shorten telomeres due to chronic exposure to reactive oxygen species (ROS). Telomeres seem to be essential for proper alignment of chromosomes and spindle integrity, and to ensure equal separation of chromosomes during meiotic cell division [13]. Interestingly, there is still controversy regarding the mechanisms underlying chromosome misalignment and disruption of spindles caused by telomere shortening with advancing maternal age of the oocyte [1, 13]. Therefore, telomere dysfunction in oocytes has been linked to reproductive senescence of woman [14, 15].

Telomeric repeat-containing RNA (TERRA) are long non-coding RNAs which are actively transcribed from the sub-telomeric region towards the telomere [16, 17]. In the last decade research concerning TERRA was intensively conducted [16, 17] with the aim to understand its function. Previous studies provided evidence that the RNA polymerase II gives rise to TERRA transcripts that contain UUAGGG-repeats. Those transcripts are also differing in size ranging from 0.2 to 10 kb in humans. [17]. TERRA microscopical appearance reflects a spotted, mainly nuclear pattern when detected by RNA-FISH [16, 17]. It has been estimated that approximately 30% of these spots co-localize with telomeres [18], what might indicate that TERRA is a component of the telomeric chromatin, although the number of spots may vary dependently on the cell type [17]. With regards to telomeres, TERRA appear to be crucial for telomere protection and stability [19–22], for telomerase recruitment and in some cases its inhibition, for telomeric heterochromatin formation, for genomic stability, and for an alternative lengthening mechanism of telomeres [23–25]. TERRA also binds to extra-telomeric places acting as an epigenomic modulator and, therefore regulating gene expression of target genes in ES cells [19]. Interestingly, TERRA regulates the transcriptional landscape of pluripotent cells through the TRF1-dependent recruitment of PRC2 [25]. The TERRA expression profile is regulated in a complex way [17, 26], and it was suggested that changes in the methylation of specific sub-telomeric CG rich islands in potential promoter regions are responsible for the earlier mentioned changes [27]. Additionally, in human somatic cells TERRA expression profiles are modulated during the cell cycle [28] and, in murine cells even during cell differentiation and development [17, 29]. With regards to reproduction, the TERRA expression was recently described in mammalian germ cells (oocytes and spermatocytes) during meiotic prophase I, where it appears to be regulated in a sex-specific manner [30, 31]. However, the role of TERRA during early embryonic development remains unclear, although initial data suggest that TERRA transcription initiates in the primordial germ cell stage [32].

According to this, and due to the fact that TERRA seems to regulate the telomeric chromatin structure, we studied the expression pattern of TERRA in early embryonic development both in bovine and murine species and with regards to maternal age. We decided to use bovine oocytes since the cow is being used as a model of human oocyte developmental competence due to numerous similarities in the reproductive physiology between the human and bovine species. Murine samples were also analyzed since they have long served as models of human biology and disease. Although both bovine and murine are good models, our results should be taken with caution when extrapolates to humans. Finally, we also measured telomere length in blood samples to understand whether there is any correlation with the observed TERRA expression.

## RESULTS

### TERRA expression profiles during early bovine embryonic cleavage stages

**Figure 1:**
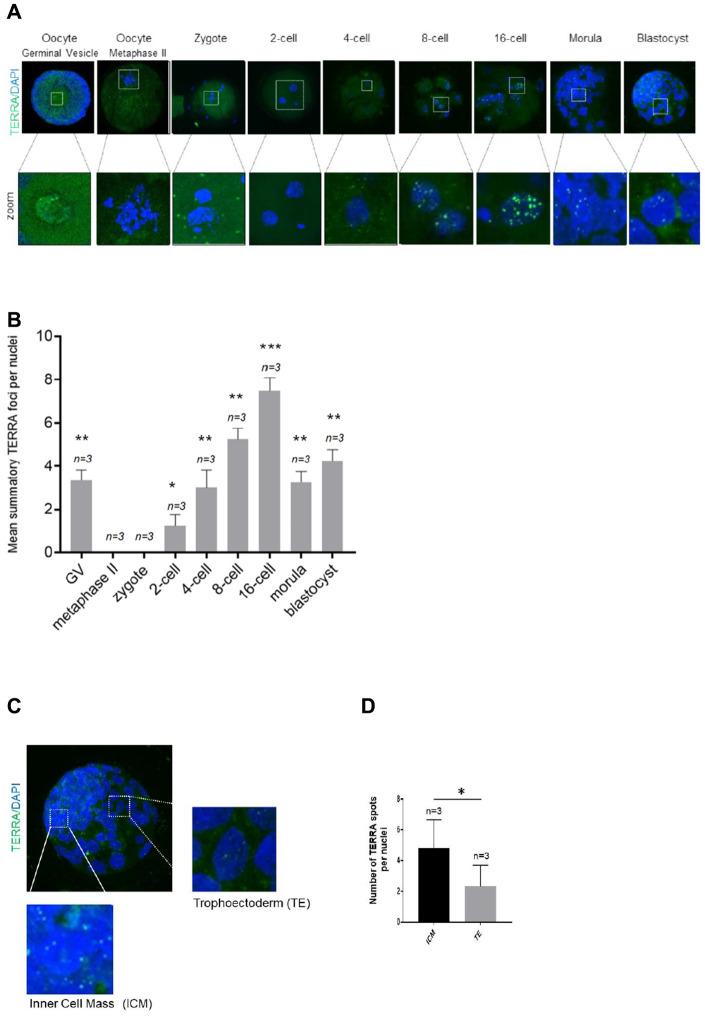
**TERRA expression in bovine oocytes, early cleavage and blastocyst stage.** (**A**) The TERRA signal was detected by RNA-FISH on bovine oocytes and different cleavage stages of bovine early embryos and blastocysts using a probe targeting TERRA’s telomeric track (green), DAPI=blue. Zoom areas are shown, where only the bright puncta correspond to TERRA transcripts (**B**). Graph shows the quantification of the mean number of TERRA spots in all developmental stages (mean+s.d., n=number of samples). (**C**) Zoom areas corresponding to the inner cell mass (ICM) and trophoectoderm (TE) are shown. (**D**) Graph represents the mean number of TERRA spots in the ICM and TE (mean+s.d., n=number of blastocysts). Total number of foci and nuclei used for the analysis are indicated. Scale bar: 10 μm. The Student’s t-test was used for all statistical analysis (*p<0.05, **p < 0.01, and ***p<0.001).

First, we set to address the pattern of TERRA during early embryonic development in the bovine species. Therefore, both TERRA levels and distribution were finally analyzed in nine stages. For this purpose, 100 bovine germinal vesicle (GV) oocytes were used for IVM, IVF and IVC to ensure a proper number of replicates of each developmental stage. Mean number of TERRA foci per nuclei (mean ±SD) in GV, metaphase oocyte, 2-cell, 4-cell, 8-cell, 16-cell, morula and blastocyst stage embryos were counted. Chi-square analysis showed that TERRA foci first decrease significantly from GV to the zygote and the 2-cell stage. Interestingly, there were no observable TERRA foci at the two latter mentioned stages (Figure 1A, 1B). It was neither observed in a RNase A-treated sample used as negative control (data not shown). Thereafter, the TERRA spots per nuclei increased significantly during early cleavage embryo development, reaching the highest levels of TERRA expression at the 16-cell stage (7.67 ±0.58) (Figure 1A, 1B). In the morula and blastocyst stage the relative TERRA level per nuclei started to show a second wave of downregulation from morula (3.33 ±0.58), and blastocyst stages (4.3 ±0.58) compared to 16-cell embryos (7.67 ±0.58) (Figure 1A, 1B). Interestingly, detailed analysis of bovine blastocysts show a significant higher TERRA expression in the cells of the inner-cell-mass (ICM) when compared to cells of the trophoectoderm (TE) (Figure 1C, 1D). Only the bright spots visualized in the zoomed regions are actual TERRA transcripts and were the only signal quantified (Figure 1C, 1D). Also note that the RNA FISH analysis tends to underrepresent copy number slightly, so the actual numbers in the quantification might be somewhat higher (Figure 1B and 1D).

### TERRA expression during early murine embryonic cleavage stages and its correlation with maternal age

**Figure 2:**
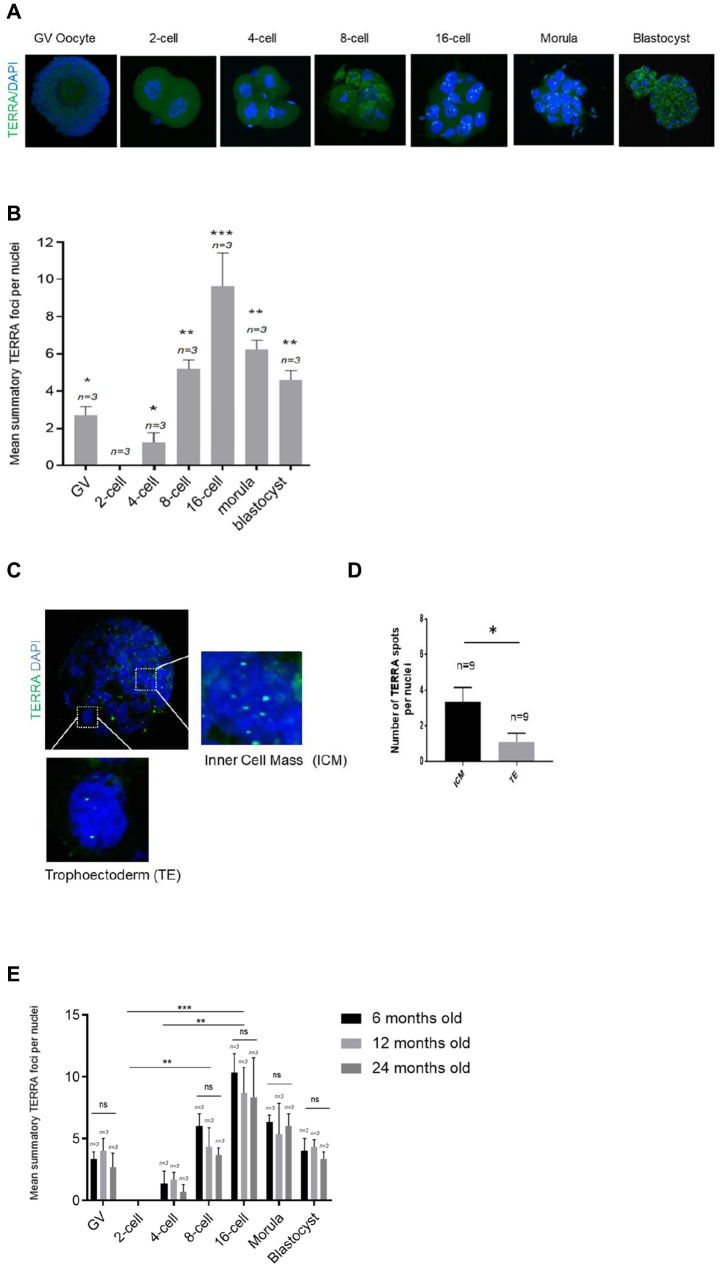
**TERRA expression in murine oocytes, early cleavage and blastocyst stage with regards to maternal age.** (**A**) The TERRA signal was detected by RNA-FISH on murine oocytes and different cleavage stages of murine early embryos and blastocysts using a probe targeting TERRA’s telomeric track (green), DAPI=blue. Zoom areas are shown, where only the bright puncta correspond to TERRA transcripts. (**B**) Graph shows the quantification of the mean number of TERRA spots in all developmental stages in the young donor group (mean+s.d., n=number of samples). (**C**) Zoom areas corresponding to the inner cell mass (ICM) and trophoectoderm (TE) are shown. (**D**) Graph represents the mean number of TERRA spots in the ICM and TE (mean+s.d., n=number of blastocysts). (**E**) Graph shows the quantification of the mean number of TERRA spots in all developmental stages in the three differently aged donor groups (6, 12 and 24 months old) (mean+s.d., n=number of samples). Total number of foci and nuclei used for the analysis are indicated. Scale bar: 10 μm. The Student’s t-test was used for all statistical analysis (*p<0.05, **p < 0.01, and ***p<0.001).

Murine TERRA levels and foci distribution were analyzed in the same developmental stages as in the bovine analysis. To do so, we used the same RNA-FISH protocol for the murine species (see Materials and Methods). For this purpose 100 murine metaphase II (MII) oocytes were used for IVF and IVC to ensure a proper number of replicates of each developmental stage. As in in the bovine species, the mean number of TERRA foci per nuclei in GV oocytes, 2-cell, 4-cell, 8-cell, 16-cell, morula and blastocyst stage embryos were evaluated. Due to the fact that the bovine oocytes and embryos were generated from heifers, meaning young donors, we first analyzed the TERRA expression on young donors for the murine species (Figure 2A, 2B). Chi-square analysis showed that TERRA foci first decrease significantly from GV to the zygote stage (Figure 2B). Interestingly, there were no observable TERRA foci at the zygote and 2-cell stage (Figure 2B), as already shown in the bovine species (Figure 1B). None foci were neither observed in a RNase A-treated sample (data not shown). Thereafter the TERRA spots per nuclei increased significantly during early cleavage embryo development representing a comparable expression pattern as shown in the bovine species (Figure 1B), reaching the highest levels of TERRA expression at the 16-cell stage (7.67 ±0.58) (Figure 2B). Likewise in the bovine embryos, the relative TERRA expression in the murine morula and blastocyst stage decreased gradually, namely in the morula (5.89 ±1.37), and blastocyst stages (4.89 ±0.78) compared to 16-cell embryos (9.11 ±2.26) (Figure 2B). As shown for the bovine blastocysts, murine counterparts as well show a significant higher TERRA expression per nucleus in the ICM cells when compared to the nuclei of the TE cells (Figure 2C, 2D). Only the bright spots visualized in the zoomed regions are actual TERRA transcripts and were the only signal quantified (Figure 2C, 2D).

Next, to test if TERRA expression is affected by the age of oocyte donor, we performed the TERRA detection in the murine species in three different age groups, namely in 6, 12 and 24 months old. Interestingly, TERRA expression did not significantly differ between the three examined age groups at any of the developmental stages studied (Figure 2E). This result indicates that TERRA expression is not influenced by the donor’s age at early stages of development of the mouse.

### Telomere length of murine oocyte donor leukocytes

**Figure 3:**
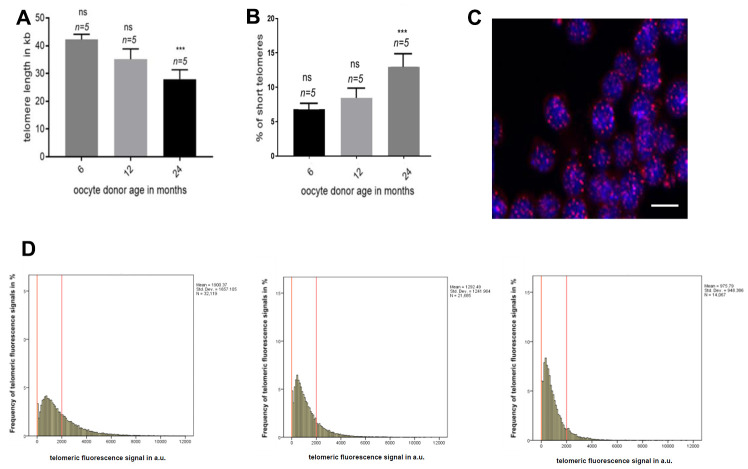
**Telomere length in oocyte donor leukocytes.** (**A**) Graph represents the quantification of the mean telomere length in the 6, 12 and 24 months old donors group by HT-Q-FISH (mean values ±s.e.m., n=number of blood samples in each group). (**B**) Graph showing the percentage of short telomeres in the same settings. Short telomeres are considered those in the 10% percentile of the total telomere length distribution (mean values ± s.e.m., n=technical replicates). (**C**) Representative confocal microscopy picture of oocyte donor leukocytes (blue=DAPI, red=telomeres, scale bar = 5um). (**D**) Representative frequency graphs of telomere length distribution in arbitrary units (a.u.) of intensity measured in the 6, 12 and 24 (from left to right) months old oocyte donors and the mean telomere length and the number of telomeres analyzed is shown. The red lines are arbitrary lines placed in the exact same position in each frequency graph to visualize differences between samples, the y-axis represents the frequency in percentage of telomere length distribution and the x-axis shows the telomeric fluorescence signal in a.u. . One-way ANOVA with Dunnett’s post test was used for the statistical analysis (*p<0.05, **p < 0.01, and ***p<0.001).

To address whether TERRA expression was influenced by telomere length, we measured telomere length in the peripheral PBMCs from donors of the same age in which TERRA was determined. The measurement was performed using the high-throughput Q-FISH (HT q-FISH) technique. We observed a significant decrease in the mean telomere length with advancing donor age (Figure 3A). Interestingly, the percentage of short telomeres, determined in all age groups as telomeres which were in the 10% percentile of the telomere length distribution, was significantly higher in PBMCs of mice with the age of 24 months when compared with the 6 and 12 months old counterparts (Figure 3B). Frequency graphs of telomere length distribution to visualize the mean telomere length and the number of telomeres which have been analyzed in the 6, 12 and 24 months old oocyte donors (Figure 3D), underline the before mentioned differences between the age groups.

## DISCUSSION

Here, we have shown that TERRA transcription starts as early as in the murine 4-cell stage reaching a maximum expression at around the 16-cell stage following a slight decline from morula to blastocyst. This wave of expression was also found in bovine samples likely indicating conservation in mammals. Moreover, TERRA expression is not affected by increasing oocyte donor age whereas telomere length in PBMCs does. This indicates that murine TERRA expression is independent of the telomere length at early stages of development in the mouse.

TERRA has been detected so far in murine primordial germ cells and human fetal ovarian cells [32, 33] but its expression in early stages of development had never been evaluated. In the present study, we found that TERRA starts transcribing as early as in the 4-cell stage and is detected until the blastocyst stage. TERRA’s presence at these stages supports an important role of TERRA during early development. This is not surprising since TERRA has been shown to exert important functions from yeast to humans. Thus, TERRA functions as an essential regulator of telomeres *in cis* and as an epigenomic modulator *in trans* at extra-telomeric sites. At telomeres TERRA participates in telomere protection and maintenance, homologous recombination, heterochromatin formation, telomere replication and regulates telomerase function [17, 20–23, 54–59]. At extra-telomeric sites, TERRA antagonizes with ATRX to regulate gene expression, which is performed through TRF1-dependent recruitment of PCR2 in pluripotent cells [19, 25]. Interestingly, TERRA increases during cellular reprograming [29]. This fact correlates well with the detection of higher number of TERRA spots in the inner cell mass than in the trophoectoderm in the bovine and murine blastocysts. In addition, it has been recently shown that there are stem cell niches in ovaries and that the immune system may help to delay ovarian aging [60]. It will be very interesting to analyse TERRA levels in this stem cells niches and the influence of the immune system in future experiments. Another interesting observation is the lack of TERRA expression at the one- and two-cell stage. At these stages, maternal mRNAs and proteins of oocytes are responsible for the very early embryonic development [34]. The clearance and degradation of maternal products which occurs until the 2-cell stage, are mandatory during the process of oocyte maturation and first embryonic cleavages [35–37]. The absence of TERRA expression might indicate that TERRA is not essential until the 4-cell stage.

Previous studies provided strong evidence that telomere alterations affect homologs’ alignment, and meiotic spindle formation [38–40], what could be related to impaired quality oocytes and therefore reflecting characteristic features of reproductive senescence in women. In case these aged oocytes are fertilized a possible outcome might be genome instability of the embryos [41]. One of the telomeric alterations observed is the telomere shortening with increasing donor age [42, 43]. Telomere length is frequently analysed in leukocytes since their telomere length is highly correlated with the telomere length in other tissues [44]. Our results concerning the telomere length in leukocytes of oocyte donors in the three age groups are in order with previous studies [42, 43]. Namely with advancing oocyte donor age (6, 12 and 24 months) we did see a decrease of telomere length in leukocytes, and the older the oocyte donor was the higher was the percentage of short telomeres. On the other hand, TERRA expression is not affected by the oocyte donor age. This indicates that murine TERRA expression at early stages of development is independent of the systemic telomere length measured in leukocytes. This is not surprising since there are many reports indicating these lack of correlation between TERRA levels and telomere length [45, 46], for instance in mouse embryonic fibroblasts or cancer ALT cell lines [47, 48]. On the contrary, TERRA has been reported to participate in the regulation of telomere length via several pathways. Firstly, TERRA can inhibit telomerase activity to promote telomere shortening [47]. Secondly, TERRA can promote Exo1-dependent resection at chromosome ends to initiate telomere shortening [47]. Nevertheless, this is a controversial issue because different results may be due to the use of different cell lines or to the different cell culturing passages which may display variations in TERRA expression [48].

The lack of correlation between TERRA expression with the oocyte donor age further stresses the idea that TERRA could be essential for mouse early development. Although, the development of the early embryo is permissive with a decrease telomere length as observed with increasing donor age, this might not be the case for TERRA. One of the facts that supports this hypothesis is the known interaction of TERRA with the Polycomb-repressive complex 2 (PRC2) [49]. PRC2 is required in epigenetic reprogramming during the early developmental stages [50]. In fact, the deletion of any of the three core PRC2 components (Ezh2, EDD and Suz12) is essential for early mouse development [51–53]. Importantly, we have recently shown that TERRA regulate the transcriptional landscape of pluripotent cells through TRF1-dependent recruitment of PRC2 [25]. All the former, supports therefore an essential role of TERRA in early development as well.

In conclusion, our findings show a first picture of TERRA expression in the early stages of embryonic development. Moreover, the TERRA detection at these critical developmental stages and its independence of oocyte donor age supports an essential role in early development. To what extend these results might be extrapolated for the human species needs to be evaluated. Further investigation will be needed to prove this when TERRA *knock-out* models are available.

## MATERIALS AND METHODS

### Mouse handling and care

All procedures performed on mice were carried out in accordance with the relevant guidelines: EU Directive of the European Parliament and the Council on the protection of animals used for scientific purposes (22 September 2010; No 2010/63/EU), Polish Parliament Act on Animal Protection (21 August 1997, Dz.U. 1997 nr 111 poz. 724) with further novelization - Polish Parliament Act on the protection of animals used for scientific or educational purposes (15 January 2015, Dz.U. 2015 poz. 266). The experimental procedures were approved by the Local Ethics Committee for Experiments on Animals, University of Warmia and Mazury in Olsztyn, Poland (Agreement No. 53/2019). Procedures were performed with 18 female wild-type mice C57BL/6 background in the age of six months (n=6), 12 months (n=6) and 24 months (n=6). Mice were produced and housed in the specific-pathogen-free animal house of PAS, Olsztyn, Poland.

### Blood collection and further processing

Blood samples were obtained via intracardiac aspiration from all 18 oocyte donors after CO_2_ euthanization on the day of cumulus-oocyte-collection. The blood samples were processed with erythrocyte lysis buffer (Qiagen cat no 79217) according to the manufacturer’s protocol and peripheral blood mononuclear cells were isolated. The cells were then frozen in 90% Fetal Bovine Serum (FBS) supplemented with 10% DMSO at -80°C slowly in a Nalgene Cryo Freezing Container (Nalgene Cat no 5100-0001) until telomere length was performed.

### Donor superovulation, oocyte collection and *in vitro* fertilization

Female mice in the age ranging from 6 to 24 months were superovulated by an intraperitoneal injection of 7.5 IU Pregnant Mare Serum Gonadotropin (PMSG, Sigma-Aldrich, Poznan, Poland) followed by 7.5 IU human chorion-gonadotropin (hCG, Sigma-Aldrich, Poznan, Poland) 48 h later. Fourteen hours after the last injection, the mice were euthanized with CO_2_ gas, and their oviducts were removed. The generated cumulus-oocyte complexes (COCs) from the ampulla of the oviduct were transferred in a 200μL drop of CARD Medium (Cosmo Bio, Tokyo, Japan) covered with silicone oil. Male mice were killed by cervical dislocation and each cauda epididymis was isolated using a pair of micro-spring scissors, and by gently pressing of the cauda epididymis with a dissecting needle the spermatozoa were released into a drop of 200μL FERTIUP medium (Cosmo Bio, Tokyo, Japan) covered with silicon oil. Thereafter, 3μL of the sperm suspension was added to the drop of CARD medium containing the COCs, and the dish was placed in a humidified incubator at 37°C, with 5% CO_2_ in air for three hours of *in vitro* fertilization. After washing in a drop of human tubal fluid medium (HTF, Cosmo Bio, Tokyo, Japan) the zygotes were placed for additional three hours of incubation at the same conditions to make the detection of the polar body possible, and to remove parthenogenetic oocytes. An overnight culture of the zygotes was performed under the same conditions as mentioned before, the obtained 2-cell stage embryos were transferred to a fresh HTF containing drop, and *in vitro* culture was continued until the blastocyst stage.

### Bovine oocyte collection, *in vitro* oocyte maturation (IVM)

Bovine cumulus–oocyte complexes (COCs) were recovered and *in vitro* matured as previously described [61]. Briefly, immature COCs were obtained by aspirating follicles (2–8 mm) from the ovaries of heifers collected at the slaughterhouse. International Embryo Transfer Society (IETS) classes I-III COCs were matured for 24 h in groups of approximately 50 COCs per well in four-well dishes (NUNC, Roskilde, Denmark) in 500 μL of *in vitro* maturation medium “IVM” (TCM 199 (M4530) supplemented with 10% (v/v) fetal calf serum (FCS) and 10 ng/mL epidermal growth factor (E4127). The culture conditions were 38.5°C, 5% CO_2_ in air and maximum humidity.

### Bovine *in vitro* fertilization (IVF)

Frozen semen from a single Holstein Frisian bull (previously tested for IVF) was thawed at 37°C in a water bath for 1 min and sperm was selected on a Bovipure gradient (Nidacon Laboratories AB, Gothenburg, Sweden) as previously described [35]. Sperm concentration was determined and adjusted to a final concentration of 1 × 10^6^ sperm cells/ mL for IVF. Gametes were co-incubated for 18 h in 500 μL of fertilization medium (Tyrode’s medium with 25 mM bicarbonate, 22 mM sodium-lactate, 1 mM sodium-pyruvate and 6 mg/mL fatty acid-free BSA) supplemented with 10 μg/mL heparin sodium salt (Calbiochem) in groups of 50 COCs per well in four-well dishes at 38.5°C in an atmosphere of 5% CO_2_ in air at maximum humidity.

### Bovine *in vitro* embryo culture (IVC)

At approximately 20 h after insemination presumptive zygotes were denuded of cumulus cells by vortexing for 3 min, randomly divided into groups of 25 and cultured in 25 μL droplets of synthetic oviductal fluid (SOF) supplemented with 4.2 mM sodium-lactate (L4263), 0.73 mM sodium pyruvate (P4562), 30 μL/mL BME amino acids (B6766), 10 μL/mL MEM non-essential amino acids (M7145) and 1 μg/mL phenol red (P0290) and 3 mg/mL bovine serum albumin (BSA; A9647). Droplets were placed under mineral oil at 38.5°C in an atmosphere of 5% CO_2_, 5% O_2_ and 90% N_2._

### High-throughput Q-FISH for telomere length measurement

To perform telomere length measurement in mouse peripheral blood mononuclear cells, blood samples were thawed quickly and re-suspended in complete RPMI media and plated in poly-L-lysine pre-coated clear bottom black-walled 96-well plates (Greiner, Kremsmünster, Upper Austria, Austria). Samples were analyzed in duplicates. The High-throughput Q-FISH (HT Q-FISH) protocol was performed as described by Canela et al. [62]. To convert telomeres fluorescence values into kb, we used standard cell lines with stable telomere length: L5178Y-R (79.7 kb), HeLa1211 (23.8 kb), Jurkat (11.5 kb), S (10.3 kb), K562 (6.5 kb), and HeLa R (6.03 kb) Images were acquired on an Opera High Content Screening System (PerkinElmer, Inc., Waltham, Massachusetts, USA) and analyzed with Acapella Image analysis software (PerkinElmer, Inc.).

### RNA-fluorescence *in situ* hybridization for TERRA’s detection

All specimens, namely oocytes, zygotes, and different embryonic stages until the blastocyst stage were washed three times in a 100uL droplet of phosphate-buffered saline (PBS) and then placed on a glass slide (SuperFrost Plus Slides, Thermo Fisher Scientific, Dreieich, Germany,) pre-treated with 0.01% poly-L-lysine (Sigma-Aldrich, Poznan, Poland) for 10min in a warming chamber at 37 °C for a proper drying and attachment. Thereafter the samples were placed in cytobuffer (100 mM NaCl, 300 mM sucrose, 3 mM MgCl2, 10 mM pipes pH 6.8) for 30 s, followed by gently washing in cytobuffer with 0.5% Triton X-100 for 30 s, and again washed in cytobuffer for 30 s. In all washing steps with the Cytobuffer, RNase- and protease inhibitors were added. After the last washing, the samples were fixed for 15 min in 4% paraformaldehyde in PBS. The cells were dehydrated in 70, 80, 95, and 100% ethanol, air-dried, and hybridized overnight at 37ºC in a humid chamber containing 50% Formamide in hybridization buffer (2 × sodium saline citrate (SSC)/50% Formamide) with a telomere-specific PNA-FITC probe (Panagene Inc., Daejeon, Repurblic of Korea). Slides were washed two times for 15 min in hybridization buffer at 39 °C, followed by one washing step for 10 min in 2 × SSC at 39 °C, 10 min in 1 × SSC at 39 °C, 5 min in 4 × SSC at RT, 5 min in 4 × SSC containing 0.1% Tween-20 at RT, and 5 min in 4 × SSC at RT. After a short air-drying step the sample’s nuclei were counterstained with ProLong Gold Antifade Mountant (Thermo Fisher Scientific, Dreieich, Germany) mounting medium and covered with a coverslip. As a negative control samples were treated 10 min with RNAse A (Sigma-Aldrich, St. Louis, MO. USA) at a concentration of 100 μg/ml prior to RNA-FISH performance. TERRA signals in were visualized in a confocal ultraspectral microscope SP5-WLL (Leica, Germany) and the TERRA spots in each image were counted. Out of the oocyte pool of all six animals in each age group, n=3 samples of each developmental stage were analyzed after performing the RNA-FISH, respectively.

### Statistical analysis

Statistical analyses were performed using GraphPad Prism version 6.00 for Macintosh (GraphPad Software, San Diego California USA, https://www.graphpad.com/). Specific analyses are described within the text and the corresponding Figures. Statistical significance was set at p value of 0.05.
